# The Effect of Novel Packaging Technology on Food Safety and Quality

**DOI:** 10.3390/foods10020269

**Published:** 2021-01-29

**Authors:** Marina Paolucci, Maria Grazia Volpe

**Affiliations:** 1 Department of Science and Technology, University of Sannio, 82100 Benevento, Italy; paolucci@unisannio.it; 2 Institute of Food Sciences, National Research Council, CNR, 83100 Avellino, Italy

This Special Issue of *Foods*, The Effect of Novel Packaging Technology on Food Safety and Quality, contains seven papers that were refereed and selected in accordance with the usual editorial standards of the journal.

It deals with results regarding innovative and creative packaging technologies with express active and intelligent functions, both in terms of shelf-life extension and quality monitoring [1,2].

The packaging technology sector is looking with growing interest at environmental sustainability, introducing recyclable and/or biodegradable materials obtained from sustainable sources to which natural bioactive substances can be added that delay oxidation, deterioration and contribute to the maintenance of the general organoleptic characteristics of the product. The evolution of new and innovative packaging techniques is strengthening the economy by reducing food losses and improving food safety and quality [3,4].

The study of Hawthorne et al. [5] reported the use of biodegradable packaging materials for the storage of fresh pork products over extended shelf-life periods, in comparison with conventional packaging materials. The research evaluated the sustainability of biodegradable packaging materials compared to well-known conventional packaging materials through analyses of the quality of freshly packaged pork in terms of color, sensory and microbiological evaluation.

The results of the sensory attributes, meat color or total bacterial count (TBC) over extended storage times showed no significant differences (*p* > 0.05) in ground pork or pork loin stored in biodegradable modified atmosphere packaging (BioMAP) or conventional MAP. On the basis of the data obtained, the authors were able to state that the use of biopolymers (BioMAP) could be a possible alternative to MAP using conventional, fossil fuel-based materials for the storage of fresh meats, and at the same time, more environmentally friendly packaging systems would be more appreciated by customers.

Bandyopadhyay et al. [6] increased the shelf life of cheese preserved in functional hydrogel able to improve its antimicrobial, physical, mechanical and thermal properties. Essential oils (EOs) were added to the hydrogel films to impart antimicrobial properties and increase hydrophobicity and mechanical properties. Moreover, the slow and steady release of EOs from the hydrogel film maintained cheese antimicrobial activity for a longer time.

The maintenance of the quality characteristics of the cheese packaged with hydrogel film (with and without EO) was successfully confirmed by the pH adhesives based on anthocyanins placed inside the packages; the cheese remained fresh for 30 days in refrigerated conditions, while the cheese in polyethylene showed decay and deterioration in quality after 18–20 days. Therefore, the results of the study allow the assertion that the hydrogel film (with and without EO) can be considered an active and functional packaging material to increase the shelf life of the cheese compared to conventional packaging.

The study of Jancikova et al. [7] focused on differences between edible packaging prepared with κ- and j-Carrageenan to assess the different textural properties between two Carrageenans—kappa carrageenan has about 25 to 30% of sulfate content and iota carrageenan about 28 to 30%, and changes in antioxidant properties due to the addition of lapacho extracts. The lapacho contains many specific polyphenols, such as caffeic, protocatechuic, p-coumaric, ferulic and also syringic acid, which allow the total polyphenol content and the antioxidant activity of the biodegradable films to be increased.

According to the reached results, the authors concluded that edible films with the highest concentrations of added lapacho extract could be a useful source of antioxidant compounds.

The results of the study will certainly serve as a useful source of information for further edible packaging formulae, as well as for the possible application to suitable types of food commodities.

The study of Martiny et al. [8] focused on carrageenan-based active packaging film prepared by adding olive leaf extract (OLE) as a bioactive agent for the preservation of lamb meat. Olive leaf extract contains many bioactive compounds, including oleuropein, verbascoside, hydroxytyrosol and tyrosol, which have antioxidant and antimicrobial properties, and have been associated with their strong characteristics as preservatives.

The addition of 62.5% (*w*/*w*) of OLE (based on carrageenan mass) to the carrageenan films significantly changed the thickness, color parameters, mechanical properties and the barrier property. However, there was no significant change in the solubility.

The film with the OLE was shown to have an antimicrobial capacity during the refrigerated storage of lamb meat, reducing the count of psychrophiles five-fold when compared to the samples packed by the control and commercial films. Overall, the carrageenan-based active packaging film extended the useful life of the lamb meat by 2 to 3 days compared to that achieved with a conventional modified atmosphere.

The results of the study allow the conclusion that this active biofilm has the potential to increase the shelf life of lamb meat, and as such, is suitable and can be used in the substitution of non-biodegradable packaging materials.

Starch-based (potato, corn, sweet potato, green bean and tapioca) edible packaging film including blueberry pomace powder (BPP) was developed by Singh et al. [9]. From blueberry juice processing wastes, including skins and seeds, it is possible to recovery vitamins; minerals; fibers; and phenolic compounds, such as anthocyanins, with antioxidant capacity and potential health benefits.

The films were characterized for optical, mechanical, thermal and physicochemical properties. Their color was not affected by the addition of BPP; particularly, when BPP was added, corn and green bean starch films showed increased light barrier properties, suitable to prevent UV radiation-induced food deterioration. The percentage of BPP added to starch had no effect on the thermal parameters of films, with the exception of the corn starch films. Finally, the average solubility of all the films made from different starch types was between 24 and 37%, which makes them suitable for applications in food products with low to intermediate moisture.

The aim of the work of Vizzini et al. [10] was to apply an active film packaging with zinc magnesium oxide nanoparticles (Zn-MgO NPs) to prolong the shelf life of sliced cold-smoked salmon (CSS) samples and develop a fast, specific and easy qPCR protocol to assess the presence of L. monocytogenes. In fact, cold-smoked salmon is considered to be of high risk of L. *monocytogenes* contamination because it does not undergo heat treatments before being consumed. The efficacy of metal oxide nanoparticles as biopreservatives in polymer-based films is probably due to the direct contact of the film with the food surface and the controlled release of the particles with antimicrobial activity onto the food surfaces.

In this study, 1 mg/mL of Zn-MgO NPs was added to the alginate film to obtain the active packaging, and it was demonstrated that the selected concentration can maintain the food product at a high level of quality for a longer period. However, the solubility and diffusion of metal ions in metal oxide nanopowders integrated into polymers and aqueous solution were shown to be low. Taking into account that no significant cytotoxic effect towards mammalian cells of Zn-MgO NPs at a concentration of <1 mg/mL was observed, the produced active packaging could be acceptable to consumers.

A sustainable approach to extend the shelf life of fresh figs by application of an edible coating able to maintain the quality of the fruit during storage was developed by Paolucci et al. [11]. The polysaccharides are very widespread and low cost, so alginate sodium and agar were used to form the edible coating. The effectiveness of coatings was enhanced with the addition of pomegranate peel extract at two different concentrations. Fresh figs in a commercial preservation system with the figs preserved in an edible coating, and an active edible coating, were analyzed to compare the impact of the conservation method on the quality of the fruit. The monitoring of qualitative parameters—pH, mechanical properties, total polyphenols and antioxidant capacity, as well as microbial load—during 15 days of shelf life showed similar values for figs in active coating to those of freshly picked fruits.

The inclusion of a component with high antioxidant and antimicrobial activity in an edible coating proved to be an excellent method for preserving the quality of this highly perishable fruit. Therefore, an active coating, obtained from renewable sources, could be used to extend the storage life of highly perishable fruits such as figs, which is often consumed in production areas due to short shelf life.

The results obtained in the various studies could be used to design active biopackaging systems with the addition of active components capable of increasing the shelf life of highly perishable products.

Eco-friendly packaging represents the answer to the collective need to reduce plastic waste, but at the same time, we propose active packaging solutions capable of safeguarding the safety and quality of foodstuffs over time. This trend in the development of packaging has arisen from the demands of consumers who prefer poorly preserved, fresh, tasty and affordable food; online purchases of transregional and transnational foods; and more frenetic lifestyles that reduce the time spent in the market and cooking.

Overall, these seven contributions published in the Special Issue further strengthen the suitable sustainable approaches for foodstuff preservation. 

Finally, we would like to thank all of the authors for their submissions, and all of the referees for their revisions and also their valuable suggestions for improving the manuscripts.
